# Crystal structure of diethyl [(4-chloro­anilino)(4-hy­droxy­phen­yl)meth­yl]phospho­nate *N*,*N*-di­methyl­formamide monosolvate

**DOI:** 10.1107/S1600536814016626

**Published:** 2014-08-01

**Authors:** Qing-Ming Wang, Ming-Juan Zhu, Jin-Ming Yang, Shan-Shan Wang, Yan-Fang Shang

**Affiliations:** a School of Pharmacy, Yancheng Teachers’ University, Yancheng, Jiangsu 224051, People’s Republic of China; b School of Chemistry and Chemical Engineering, Nantong University, Nantong, JiangSu 226000, People’s Republic of China

**Keywords:** crystal structure, hydrogen bond, phospho­nate

## Abstract

In the title compound, C_17_H_21_ClNO_4_P·C_3_H_7_NO, the dihedral angle formed by the aromatic rings is 83.98 (7)°. In the crystal, O—H⋯O, N—H⋯O and C—H⋯O hydrogen bonds link the mol­ecules into double layers parallel to (011).

## Related literature   

For background to the synthesis and properties of α-amino­phospho­nic acids, see: Puius *et al.* (1997 ▶); Hum *et al.* (2002 ▶); Evindar *et al.* (2009 ▶); Meyer *et al.* (2004 ▶); Kachkovskyi & Kolodiazhnyi (2007 ▶); Sieńczyk & Oleksyszyn (2009 ▶). For the structures of related compounds, see: Li *et al.* (2008 ▶); Wang *et al.* (2012 ▶).
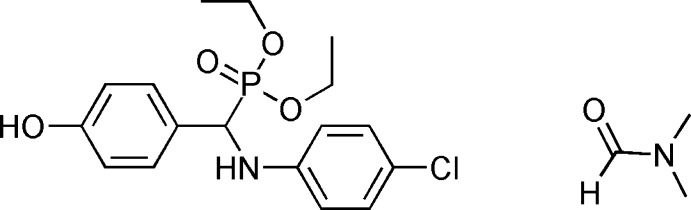


## Experimental   

### Crystal data  

C_17_H_21_ClNO_4_P·C_3_H_7_NO
*M* *_r_* = 442.86
Triclinic, 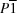

*a* = 7.7230 (3) Å
*b* = 11.6834 (5) Å
*c* = 13.4582 (5) Å
α = 69.872 (2)°
β = 88.159 (2)°
γ = 83.841 (2)°
*V* = 1133.58 (8) Å^3^
*Z* = 2
Mo *K*α radiation
μ = 0.27 mm^−1^
*T* = 296 K
0.40 × 0.20 × 0.15 mm

### Data collection  

Bruker SMART CCD area-detector diffractometer
Absorption correction: multi-scan (*SADABS*; Bruker, 2000 ▶) *T*_min_ = 0.937, *T*_max_ = 0.960
17340 measured reflections
5126 independent reflections
3982 reflections with *I* > 2σ(*I*)
*R*_int_ = 0.022

### Refinement  

*R*[*F* ^2^ > 2σ(*F* ^2^)] = 0.057
*wR*(*F* ^2^) = 0.174
*S* = 1.04
5126 reflections
271 parameters
1 restraint
H atoms treated by a mixture of independent and constrained refinement
Δρ_max_ = 0.41 e Å^−3^
Δρ_min_ = −0.41 e Å^−3^

### 

Data collection: *SMART* (Bruker, 2000 ▶); cell refinement: *SAINT* (Bruker, 2000 ▶); data reduction: *SAINT*; program(s) used to solve structure: *SHELXS97* (Sheldrick, 2008 ▶); program(s) used to refine structure: *SHELXL97* (Sheldrick, 2008 ▶); molecular graphics: *SHELXTL/PC* (Sheldrick, 2008 ▶); software used to prepare material for publication: *SHELXL97*.

## Supplementary Material

Crystal structure: contains datablock(s) I, New_Global_Publ_Block. DOI: 10.1107/S1600536814016626/rz5129sup1.cif

Structure factors: contains datablock(s) I. DOI: 10.1107/S1600536814016626/rz5129Isup2.hkl

Supporting information file. DOI: 10.1107/S1600536814016626/rz5129Isup3.cml

**Fig. 1.**
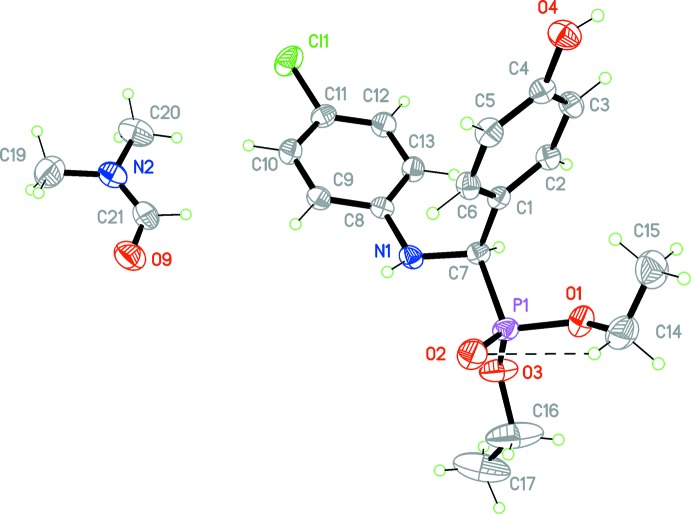
The molecular structure of the title compound with displacement ellipsoids drawn at the 30% probability level. An intramolecular hydrogen bond is shown as a dashed line.

. DOI: 10.1107/S1600536814016626/rz5129fig1.tif
The mol­ecular structure of the title compound with displacement ellipsoids drawn at the 30% probability level. An intra­molecular hydrogen bond is shown as a dashed line.

**Fig. 2.**
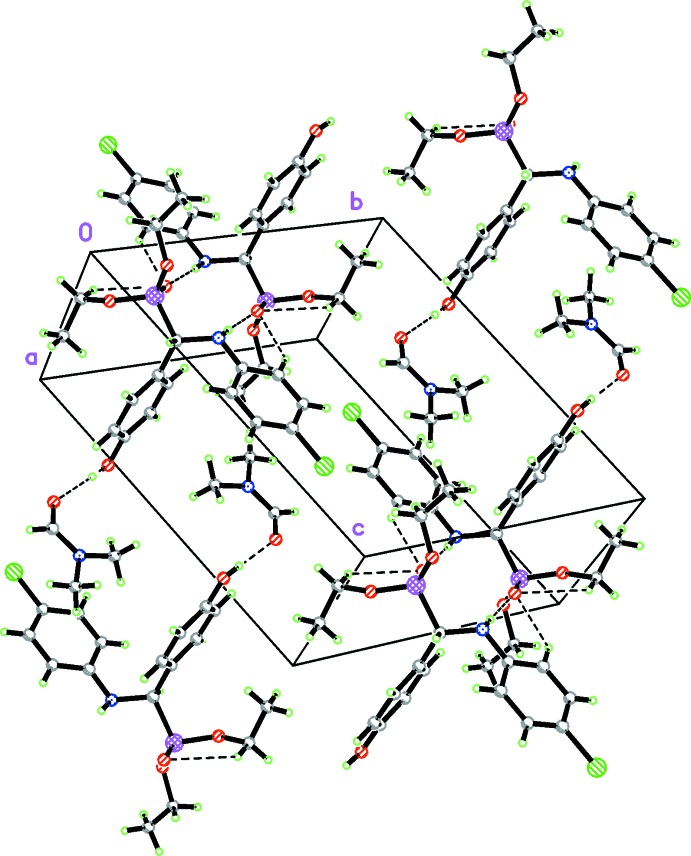
Partial crystal packing of the title compound showing the intra- and intermolecular hydrogen bonding network (dashed lines).

. DOI: 10.1107/S1600536814016626/rz5129fig2.tif
Partial crystal packing of the title compound showing the intra- and inter­molecular hydrogen bonding network (dashed lines).

CCDC reference: 1014609

Additional supporting information:  crystallographic information; 3D view; checkCIF report

## Figures and Tables

**Table 1:** Hydrogen-bond geometry (Å, °)

*D*—H⋯*A*	*D*—H	H⋯*A*	*D*⋯*A*	*D*—H⋯*A*
O4—H4⋯O9^i^	0.82	1.87	2.693 (3)	176
N1—H1*A*⋯O2^ii^	0.79 (3)	2.19 (3)	2.977 (3)	174 (3)
C7—H7⋯O4^iii^	0.98	2.53	3.502 (3)	172
C9—H13⋯O2^ii^	0.93	2.54	3.304 (3)	140

Symmetry codes: (i) 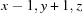; (ii) 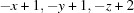; (iii) 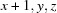.

## supplementary crystallographic information

### S1. Comment

α-Aminophosphonic acids and relative derivatives are currently attracting a great deal of interest because of their growing applications in medicine and agriculture. It has been reported that these type of compounds have antibacterial, anticancer, antibacterial, and enzyme inhibitory properties (Puius *et al.*, 1997; Hum *et al.*, 2002; Evindar *et al.*, 2009; Meyer *et al.*, 2004), and since now many α-aminophosphonic acids have been synthesized and characterized due to these reasons (Kachkovskyi & Kolodiazhnyi, 2007; Sieńczyk & Oleksyszyn, 2009). As a further contribution to this research field, the title compound was synthesized and its crystal structure is described herein.

In the title compound (Fig. 1), the P1 atom has a distorted tetrahedral geometry involving two O atoms from ethoxy groups (O1, O3), one C_α_ atom (C7), and a doubly-bonded O atom(O2). The C_α_ atom is chiral. The C–P and P═O bond lengths are comparable with those reported for similar structures (Li *et al.*, 2008, Wang *et al.*, 2012). The dihedral angle formed by the aromatic rings is 83.98 (7)°. The molecular conformation is stabilized by an intramolecular C—H···O hydrogen bond (Table 1). In the crystal structure, the molecules interact through O–H···O, N–H···O and C–H···O hydrogen bonds to form double layers parallel to the (0 1 1) plane (Fig. 2, Table 1).

### S2. Experimental

The title compound was synthesized according to a recently reported procedure (Wang *et al.*, 2012). 4-Chlorobenzenamine (0.64 g) and 4-hydroxybenzaldehyde (0.61 g) were mixed in 20.0 mL ethanol and refluxed for 1 h, then cooled to room temperature. The light yellow solid obtained was separated and washed with ethanol and ether. Part of the solid (0.462 g) was mixed with 300 mL diethyl phosphonate in 15 mL ethanol, and the mixture refluxed for 24 h. After cooling to room temperature, the light yellow oil obtained was dissolved in 10 mL DMF. Block yellow crystals of the title compound formed from the filtrate on slow evaporation of the solvent in air after two weeks.

### S3. Refinement

The amine H atom was located in a difference Fourier map and refined freely. All other H atoms were placed in geometrically idealized positions and refined as riding, with C–H = 0.93-0.97 Å, O–H = 0.82 Å, and with *U*_iso_(H) = 1.2 *U*_eq_(C) or 1.5 *U*_eq_(C, O) for hydroxyl and methyl H atoms. A rotating model was used for the hydroxyl and methyl groups. During the refinement, the C16–C17 bond length was constrained to be 1.54 (1) Å. 13 Outliers were omitted in the last cycles of refinement.

### Crystal data

**Table:**

C_17_H_21_ClNO_4_P·C_3_H_7_NO	*Z* = 2
*M**_r_* = 442.86	*F*(000) = 468
Triclinic, *P*1	*D*_x_ = 1.297 Mg m^−^^3^
Hall symbol: -P 1	Mo *K*α radiation, λ = 0.71073 Å
*a* = 7.7230 (3) Å	Cell parameters from 9970 reflections
*b* = 11.6834 (5) Å	θ = 2.7–27.5°
*c* = 13.4582 (5) Å	µ = 0.27 mm^−^^1^
α = 69.872 (2)°	*T* = 296 K
β = 88.159 (2)°	Block, yellow
γ = 83.841 (2)°	0.40 × 0.20 × 0.15 mm
*V* = 1133.58 (8) Å^3^	

### Data collection

**Table:**

Bruker SMART CCD area-detector diffractometer	5126 independent reflections
Radiation source: fine-focus sealed tube	3982 reflections with *I* > 2σ(*I*)
Graphite monochromator	*R*_int_ = 0.022
phi and ω scans	θ_max_ = 27.6°, θ_min_ = 3.5°
Absorption correction: multi-scan (*SADABS*; Bruker, 2000)	*h* = −10→9
*T*_min_ = 0.937, *T*_max_ = 0.960	*k* = −15→13
17340 measured reflections	*l* = −17→17

### Refinement

**Table:**

Refinement on *F*^2^	Primary atom site location: structure-invariant direct methods
Least-squares matrix: full	Secondary atom site location: difference Fourier map
*R*[*F*^2^ > 2σ(*F*^2^)] = 0.057	Hydrogen site location: inferred from neighbouring sites
*wR*(*F*^2^) = 0.174	H atoms treated by a mixture of independent and constrained refinement
*S* = 1.04	*w* = 1/[σ^2^(*F*_o_^2^) + (0.0923*P*)^2^ + 0.5091*P*] where *P* = (*F*_o_^2^ + 2*F*_c_^2^)/3
5126 reflections	(Δ/σ)_max_ = 0.001
271 parameters	Δρ_max_ = 0.41 e Å^−^^3^
1 restraint	Δρ_min_ = −0.41 e Å^−^^3^

### Special details

**Table:**

Geometry. All e.s.d.'s (except the e.s.d. in the dihedral angle between two l.s. planes) are estimated using the full covariance matrix. The cell e.s.d.'s are taken into account individually in the estimation of e.s.d.'s in distances, angles and torsion angles; correlations between e.s.d.'s in cell parameters are only used when they are defined by crystal symmetry. An approximate (isotropic) treatment of cell e.s.d.'s is used for estimating e.s.d.'s involving l.s. planes.
Refinement. Refinement of *F*^2^ against ALL reflections. The weighted *R*-factor *wR* and goodness of fit *S* are based on *F*^2^, conventional *R*-factors *R* are based on *F*, with *F* set to zero for negative *F*^2^. The threshold expression of *F*^2^ > σ(*F*^2^) is used only for calculating *R*-factors(gt) *etc*. and is not relevant to the choice of reflections for refinement. *R*-factors based on *F*^2^ are statistically about twice as large as those based on *F*, and *R*- factors based on ALL data will be even larger.

### Fractional atomic coordinates and isotropic or equivalent isotropic displacement parameters (Å^2^)

**Table:**

	*x*	*y*	*z*	*U*_iso_*/*U*_eq_	
C1	0.3710 (3)	0.80977 (18)	0.82727 (15)	0.0377 (4)	
C2	0.3340 (3)	0.93464 (19)	0.77767 (17)	0.0432 (5)	
H2	0.4242	0.9847	0.7633	0.052*	
C3	0.0306 (3)	0.9130 (2)	0.76955 (17)	0.0449 (5)	
C4	0.1652 (3)	0.9867 (2)	0.74897 (18)	0.0473 (5)	
H4A	0.1425	1.0710	0.7160	0.057*	
C5	0.0659 (3)	0.7874 (2)	0.81811 (19)	0.0486 (5)	
H5	−0.0241	0.7373	0.8317	0.058*	
C6	0.2337 (3)	0.73708 (19)	0.84612 (18)	0.0456 (5)	
H6	0.2561	0.6527	0.8784	0.055*	
C7	0.5553 (3)	0.75408 (18)	0.86193 (15)	0.0394 (4)	
H7	0.6331	0.8180	0.8305	0.047*	
C8	0.6226 (2)	0.66491 (19)	0.72271 (16)	0.0394 (4)	
C9	0.6075 (3)	0.5653 (2)	0.69055 (18)	0.0459 (5)	
H13	0.5904	0.4897	0.7410	0.055*	
C10	0.6176 (3)	0.5765 (2)	0.58476 (19)	0.0508 (5)	
H9	0.6067	0.5091	0.5645	0.061*	
C11	0.6438 (3)	0.6878 (2)	0.50973 (17)	0.0499 (5)	
C12	0.6600 (3)	0.7882 (2)	0.53900 (18)	0.0534 (6)	
H11	0.6782	0.8631	0.4879	0.064*	
C13	0.6491 (3)	0.7771 (2)	0.64451 (18)	0.0487 (5)	
H12	0.6595	0.8452	0.6639	0.058*	
C14	0.3741 (5)	0.8376 (4)	1.1019 (2)	0.0828 (9)	
H14A	0.3369	0.7573	1.1394	0.099*	
H14B	0.4171	0.8685	1.1537	0.099*	
C15	0.2261 (5)	0.9194 (3)	1.0463 (3)	0.0879 (10)	
H15A	0.2638	0.9978	1.0060	0.132*	
H15B	0.1401	0.9292	1.0966	0.132*	
H15C	0.1767	0.8853	0.9995	0.132*	
C16	0.8504 (6)	0.6584 (6)	1.1231 (3)	0.144 (2)	
H16A	0.8621	0.7317	1.1397	0.173*	
H16B	0.7723	0.6098	1.1745	0.173*	
C17	1.0151 (6)	0.5904 (5)	1.1311 (4)	0.150 (2)	
H17A	1.0022	0.5138	1.1219	0.225*	
H17B	1.0648	0.5751	1.1995	0.225*	
H17C	1.0904	0.6361	1.0772	0.225*	
C19	0.8263 (6)	0.1600 (4)	0.4743 (3)	0.0995 (11)	
H19A	0.9352	0.1152	0.4677	0.149*	
H19B	0.7566	0.1764	0.4122	0.149*	
H19C	0.7658	0.1123	0.5355	0.149*	
C20	0.8875 (5)	0.3742 (4)	0.3889 (3)	0.0956 (11)	
H20A	0.9054	0.4455	0.4057	0.143*	
H20B	0.7877	0.3919	0.3433	0.143*	
H20C	0.9884	0.3515	0.3537	0.143*	
C21	0.8634 (4)	0.2850 (3)	0.5803 (3)	0.0739 (8)	
H21	0.8868	0.3608	0.5818	0.089*	
Cl1	0.65380 (12)	0.70206 (8)	0.37615 (5)	0.0787 (3)	
N1	0.6162 (3)	0.65004 (18)	0.82953 (14)	0.0452 (4)	
H1A	0.589 (3)	0.584 (3)	0.862 (2)	0.051 (7)*	
N2	0.8584 (3)	0.2738 (2)	0.4857 (2)	0.0673 (6)	
O1	0.5143 (2)	0.82636 (17)	1.02988 (14)	0.0611 (5)	
O2	0.4857 (3)	0.60211 (17)	1.06464 (14)	0.0713 (6)	
O3	0.7772 (3)	0.6924 (2)	1.01916 (15)	0.0798 (6)	
O4	−0.1380 (2)	0.95971 (17)	0.74345 (16)	0.0611 (5)	
H4	−0.1433	1.0346	0.7171	0.092*	
O9	0.8404 (3)	0.2062 (2)	0.66580 (19)	0.0866 (7)	
P1	0.57543 (8)	0.70788 (5)	1.00447 (4)	0.04728 (19)	

### Atomic displacement parameters (Å^2^)

**Table:**

	*U*^11^	*U*^22^	*U*^33^	*U*^12^	*U*^13^	*U*^23^
C1	0.0417 (10)	0.0406 (10)	0.0315 (9)	−0.0111 (8)	0.0061 (7)	−0.0118 (8)
C2	0.0443 (11)	0.0399 (10)	0.0437 (11)	−0.0144 (8)	0.0053 (8)	−0.0094 (9)
C3	0.0425 (11)	0.0519 (12)	0.0413 (11)	−0.0078 (9)	0.0032 (8)	−0.0167 (9)
C4	0.0526 (12)	0.0361 (10)	0.0484 (12)	−0.0072 (9)	0.0033 (9)	−0.0078 (9)
C5	0.0446 (12)	0.0495 (12)	0.0526 (12)	−0.0193 (9)	0.0080 (9)	−0.0150 (10)
C6	0.0527 (12)	0.0344 (10)	0.0473 (12)	−0.0129 (9)	0.0071 (9)	−0.0092 (9)
C7	0.0430 (11)	0.0397 (10)	0.0319 (9)	−0.0105 (8)	0.0056 (8)	−0.0064 (8)
C8	0.0341 (10)	0.0431 (11)	0.0374 (10)	−0.0031 (8)	0.0043 (7)	−0.0096 (8)
C9	0.0459 (11)	0.0419 (11)	0.0443 (11)	−0.0073 (9)	0.0040 (9)	−0.0073 (9)
C10	0.0540 (13)	0.0508 (13)	0.0513 (13)	−0.0111 (10)	0.0028 (10)	−0.0208 (10)
C11	0.0511 (12)	0.0614 (14)	0.0377 (11)	−0.0103 (10)	0.0040 (9)	−0.0165 (10)
C12	0.0669 (15)	0.0486 (12)	0.0400 (11)	−0.0150 (11)	0.0088 (10)	−0.0073 (9)
C13	0.0624 (14)	0.0416 (11)	0.0415 (11)	−0.0117 (10)	0.0084 (10)	−0.0122 (9)
C14	0.094 (2)	0.101 (2)	0.0568 (16)	0.0018 (19)	0.0125 (15)	−0.0368 (17)
C15	0.093 (2)	0.089 (2)	0.083 (2)	0.0007 (19)	0.0159 (19)	−0.0359 (19)
C16	0.090 (3)	0.240 (6)	0.073 (3)	0.012 (3)	−0.026 (2)	−0.021 (3)
C17	0.089 (3)	0.175 (5)	0.120 (4)	−0.013 (3)	−0.028 (3)	0.036 (3)
C19	0.126 (3)	0.098 (3)	0.082 (2)	−0.023 (2)	−0.005 (2)	−0.035 (2)
C20	0.089 (2)	0.089 (2)	0.083 (2)	−0.0010 (19)	0.0005 (19)	0.0003 (19)
C21	0.0759 (19)	0.0589 (16)	0.084 (2)	0.0154 (14)	−0.0077 (16)	−0.0272 (16)
Cl1	0.1139 (6)	0.0865 (5)	0.0423 (3)	−0.0296 (4)	0.0097 (3)	−0.0257 (3)
N1	0.0548 (11)	0.0377 (10)	0.0362 (9)	−0.0040 (8)	0.0068 (8)	−0.0046 (7)
N2	0.0594 (13)	0.0660 (14)	0.0676 (15)	0.0037 (10)	0.0001 (11)	−0.0146 (12)
O1	0.0711 (11)	0.0623 (11)	0.0590 (10)	−0.0183 (9)	0.0095 (8)	−0.0298 (9)
O2	0.1158 (16)	0.0534 (10)	0.0411 (9)	−0.0282 (10)	0.0133 (9)	−0.0070 (8)
O3	0.0603 (12)	0.1195 (18)	0.0472 (10)	0.0072 (11)	−0.0112 (8)	−0.0166 (11)
O4	0.0439 (9)	0.0629 (11)	0.0720 (12)	−0.0056 (7)	−0.0031 (8)	−0.0172 (9)
O9	0.1075 (17)	0.0732 (14)	0.0685 (14)	0.0198 (12)	0.0008 (12)	−0.0195 (11)
P1	0.0558 (4)	0.0495 (3)	0.0333 (3)	−0.0099 (3)	0.0030 (2)	−0.0090 (2)

### Geometric parameters (Å, º)

**Table:**

C1—C2	1.381 (3)	C14—H14A	0.9700
C1—C6	1.392 (3)	C14—H14B	0.9700
C1—C7	1.517 (3)	C15—H15A	0.9600
C2—C4	1.386 (3)	C15—H15B	0.9600
C2—H2	0.9300	C15—H15C	0.9600
C3—O4	1.365 (3)	C16—C17	1.412 (6)
C3—C4	1.381 (3)	C16—O3	1.434 (4)
C3—C5	1.386 (3)	C16—H16A	0.9700
C4—H4A	0.9300	C16—H16B	0.9700
C5—C6	1.372 (3)	C17—H17A	0.9600
C5—H5	0.9300	C17—H17B	0.9600
C6—H6	0.9300	C17—H17C	0.9600
C7—N1	1.455 (3)	C19—N2	1.438 (4)
C7—P1	1.812 (2)	C19—H19A	0.9600
C7—H7	0.9800	C19—H19B	0.9600
C8—N1	1.387 (3)	C19—H19C	0.9600
C8—C9	1.391 (3)	C20—N2	1.453 (4)
C8—C13	1.401 (3)	C20—H20A	0.9600
C9—C10	1.384 (3)	C20—H20B	0.9600
C9—H13	0.9300	C20—H20C	0.9600
C10—C11	1.375 (3)	C21—O9	1.222 (4)
C10—H9	0.9300	C21—N2	1.326 (4)
C11—C12	1.379 (3)	C21—H21	0.9300
C11—Cl1	1.747 (2)	N1—H1A	0.79 (3)
C12—C13	1.381 (3)	O1—P1	1.5592 (18)
C12—H11	0.9300	O2—P1	1.4568 (18)
C13—H12	0.9300	O3—P1	1.560 (2)
C14—C15	1.452 (5)	O4—H4	0.8200
C14—O1	1.455 (3)		
			
C2—C1—C6	117.98 (19)	C14—C15—H15A	109.5
C2—C1—C7	120.88 (17)	C14—C15—H15B	109.5
C6—C1—C7	121.12 (18)	H15A—C15—H15B	109.5
C1—C2—C4	121.24 (19)	C14—C15—H15C	109.5
C1—C2—H2	119.4	H15A—C15—H15C	109.5
C4—C2—H2	119.4	H15B—C15—H15C	109.5
O4—C3—C4	122.1 (2)	C17—C16—O3	111.6 (4)
O4—C3—C5	118.2 (2)	C17—C16—H16A	109.3
C4—C3—C5	119.7 (2)	O3—C16—H16A	109.3
C3—C4—C2	119.8 (2)	C17—C16—H16B	109.3
C3—C4—H4A	120.1	O3—C16—H16B	109.3
C2—C4—H4A	120.1	H16A—C16—H16B	108.0
C6—C5—C3	119.93 (19)	C16—C17—H17A	109.5
C6—C5—H5	120.0	C16—C17—H17B	109.5
C3—C5—H5	120.0	H17A—C17—H17B	109.5
C5—C6—C1	121.4 (2)	C16—C17—H17C	109.5
C5—C6—H6	119.3	H17A—C17—H17C	109.5
C1—C6—H6	119.3	H17B—C17—H17C	109.5
N1—C7—C1	114.86 (17)	N2—C19—H19A	109.5
N1—C7—P1	108.79 (13)	N2—C19—H19B	109.5
C1—C7—P1	110.17 (13)	H19A—C19—H19B	109.5
N1—C7—H7	107.6	N2—C19—H19C	109.5
C1—C7—H7	107.6	H19A—C19—H19C	109.5
P1—C7—H7	107.6	H19B—C19—H19C	109.5
N1—C8—C9	119.85 (19)	N2—C20—H20A	109.5
N1—C8—C13	122.3 (2)	N2—C20—H20B	109.5
C9—C8—C13	117.79 (19)	H20A—C20—H20B	109.5
C10—C9—C8	121.2 (2)	N2—C20—H20C	109.5
C10—C9—H13	119.4	H20A—C20—H20C	109.5
C8—C9—H13	119.4	H20B—C20—H20C	109.5
C11—C10—C9	119.7 (2)	O9—C21—N2	126.9 (3)
C11—C10—H9	120.1	O9—C21—H21	116.5
C9—C10—H9	120.1	N2—C21—H21	116.5
C10—C11—C12	120.5 (2)	C8—N1—C7	119.49 (17)
C10—C11—Cl1	119.60 (19)	C8—N1—H1A	109 (2)
C12—C11—Cl1	119.88 (18)	C7—N1—H1A	120.2 (19)
C11—C12—C13	119.8 (2)	C21—N2—C19	121.2 (3)
C11—C12—H11	120.1	C21—N2—C20	122.2 (3)
C13—C12—H11	120.1	C19—N2—C20	116.6 (3)
C12—C13—C8	121.0 (2)	C14—O1—P1	125.2 (2)
C12—C13—H12	119.5	C16—O3—P1	119.8 (2)
C8—C13—H12	119.5	C3—O4—H4	109.5
C15—C14—O1	111.8 (3)	O2—P1—O1	114.07 (11)
C15—C14—H14A	109.2	O2—P1—O3	115.77 (13)
O1—C14—H14A	109.2	O1—P1—O3	104.04 (12)
C15—C14—H14B	109.2	O2—P1—C7	115.27 (11)
O1—C14—H14B	109.2	O1—P1—C7	104.53 (10)
H14A—C14—H14B	107.9	O3—P1—C7	101.57 (10)
			
C6—C1—C2—C4	1.1 (3)	N1—C8—C13—C12	178.1 (2)
C7—C1—C2—C4	−177.80 (19)	C9—C8—C13—C12	−0.1 (3)
O4—C3—C4—C2	179.6 (2)	C9—C8—N1—C7	−152.9 (2)
C5—C3—C4—C2	−0.5 (3)	C13—C8—N1—C7	29.0 (3)
C1—C2—C4—C3	−0.3 (3)	C1—C7—N1—C8	59.6 (2)
O4—C3—C5—C6	−179.6 (2)	P1—C7—N1—C8	−176.39 (16)
C4—C3—C5—C6	0.5 (3)	O9—C21—N2—C19	−1.1 (5)
C3—C5—C6—C1	0.3 (3)	O9—C21—N2—C20	179.8 (3)
C2—C1—C6—C5	−1.1 (3)	C15—C14—O1—P1	−112.5 (3)
C7—C1—C6—C5	177.8 (2)	C17—C16—O3—P1	150.0 (4)
C2—C1—C7—N1	−130.6 (2)	C14—O1—P1—O2	−3.4 (3)
C6—C1—C7—N1	50.6 (2)	C14—O1—P1—O3	−130.5 (2)
C2—C1—C7—P1	106.19 (19)	C14—O1—P1—C7	123.4 (2)
C6—C1—C7—P1	−72.6 (2)	C16—O3—P1—O2	−55.8 (4)
N1—C8—C9—C10	−178.4 (2)	C16—O3—P1—O1	70.2 (4)
C13—C8—C9—C10	−0.3 (3)	C16—O3—P1—C7	178.5 (4)
C8—C9—C10—C11	0.3 (3)	N1—C7—P1—O2	−56.14 (19)
C9—C10—C11—C12	0.0 (4)	C1—C7—P1—O2	70.60 (18)
C9—C10—C11—Cl1	−179.24 (18)	N1—C7—P1—O1	177.83 (14)
C10—C11—C12—C13	−0.3 (4)	C1—C7—P1—O1	−55.43 (16)
Cl1—C11—C12—C13	178.91 (19)	N1—C7—P1—O3	69.84 (17)
C11—C12—C13—C8	0.3 (4)	C1—C7—P1—O3	−163.43 (15)

### Hydrogen-bond geometry (Å, º)

**Table:**

*D*—H···*A*	*D*—H	H···*A*	*D*···*A*	*D*—H···*A*
O4—H4···O9^i^	0.82	1.87	2.693 (3)	176
N1—H1*A*···O2^ii^	0.79 (3)	2.19 (3)	2.977 (3)	174 (3)
C7—H7···O4^iii^	0.98	2.53	3.502 (3)	172
C9—H13···O2^ii^	0.93	2.54	3.304 (3)	140

Symmetry codes: (i) *x*−1, *y*+1, *z*; (ii) −*x*+1, −*y*+1, −*z*+2; (iii) *x*+1, *y*, *z*.
