# Supplementary figures and images for: Crystal structure of diethyl [(4-chloro­anilino)(4-hy­droxy­phen­yl)meth­yl]phospho­nate N,N-di­methyl­formamide monosolvate
Source: Acta Crystallogr Sect E Struct Rep Online. 2014 Aug 1;70(Pt 9):o919–20. doi: 10.1107/S1600536814016626 (PMC4186167; doi:10.1107/S1600536814016626)

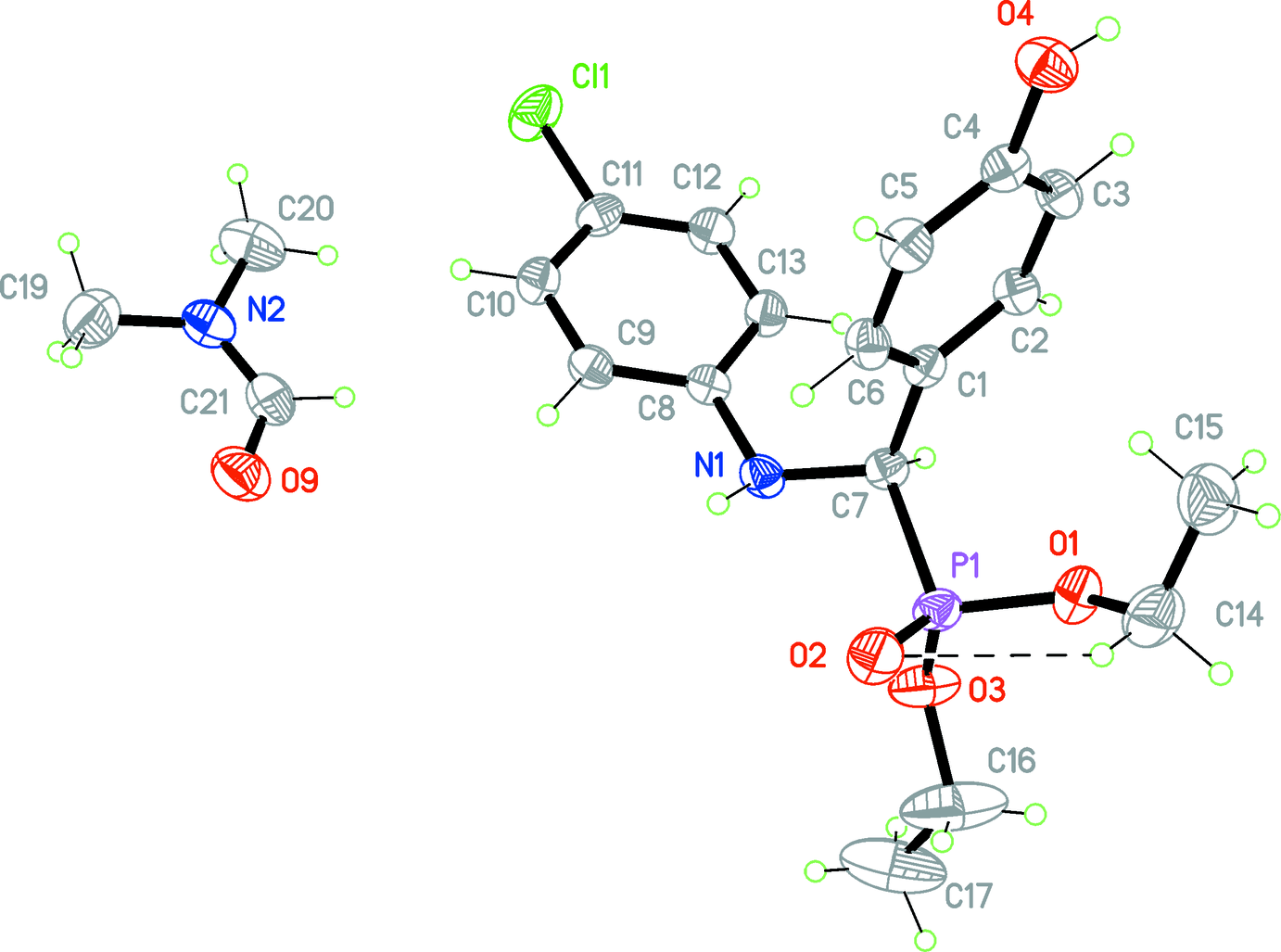

Supplement: Supplementary file 4 [file e-70-0o919-fig1.tif]

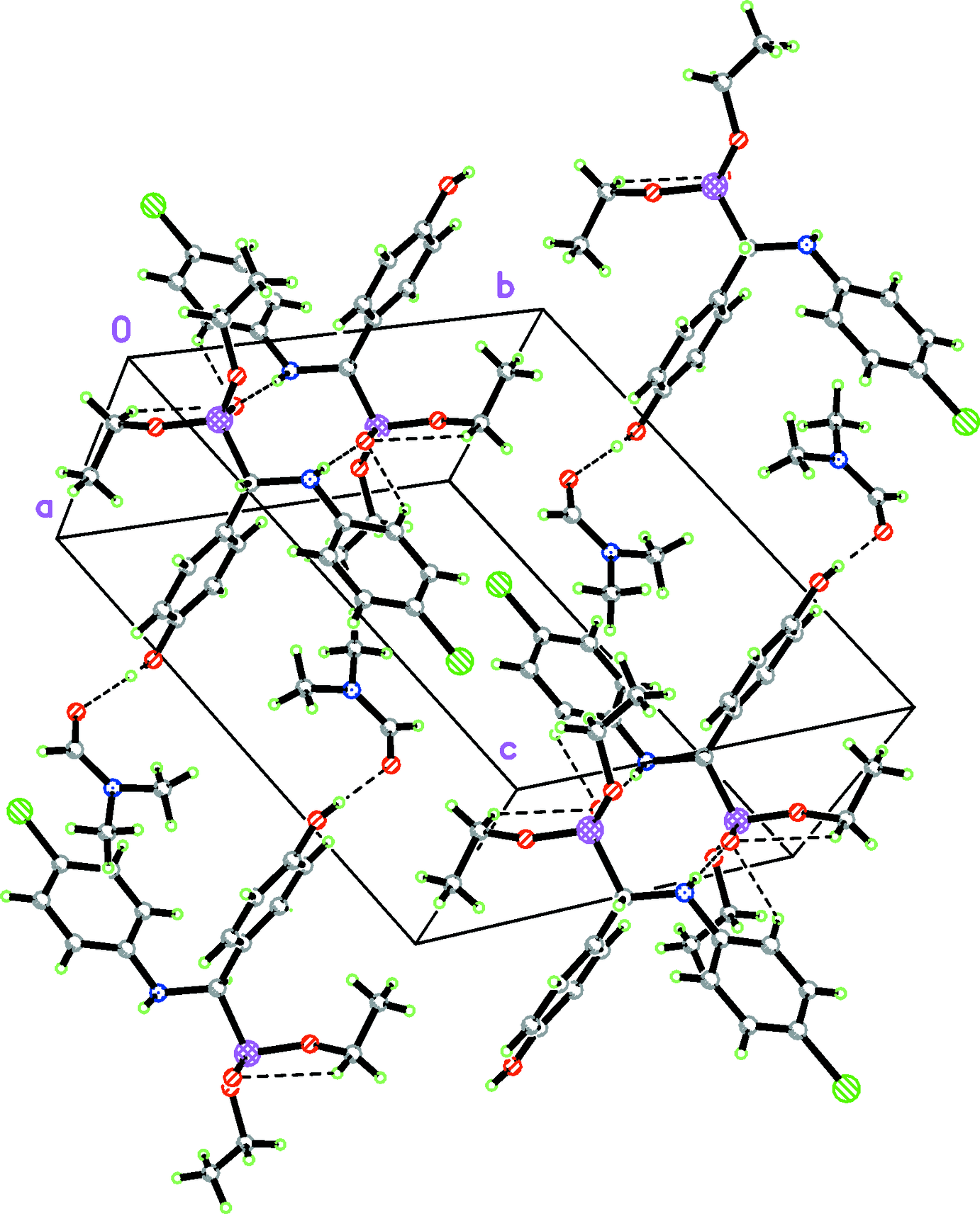

Supplement: Supplementary file 5 [file e-70-0o919-fig2.tif]
